# Potential of Metabolic MRI to Address Unmet Clinical Needs in Localised Kidney Cancer

**DOI:** 10.3390/cancers17111773

**Published:** 2025-05-26

**Authors:** Ines Horvat-Menih, Grant D. Stewart, Ferdia A. Gallagher

**Affiliations:** 1 Department of Radiology, University of Cambridge, Cambridge CB2 0QQ, UK; ih357@cam.ac.uk; 2 Department of Surgery, University of Cambridge, Cambridge CB2 0QQ, UK; gds35@cam.ac.uk

**Keywords:** renal cell carcinoma, renal oncocytoma, renal mass biopsy, treatment response, metabolic heterogeneity, hyperpolarised [1-^13^C]pyruvate MRI, deuterium metabolic imaging, sodium MRI

## Abstract

Renal cell carcinoma (RCC) is a major global health issue with an increasing incidence and mortality rate. Current diagnostic methods are either invasive or limited in their ability to accurately differentiate between benign and malignant tumours and to predict early treatment response. This can lead to incorrect diagnosis, delayed treatment, patient anxiety, and suboptimal outcomes. RCC subtypes are known to exhibit distinct metabolic alterations, for example in glucose metabolism. These metabolic phenotypes offer potential targets for non-invasive imaging techniques to improve diagnosis and treatment, but current clinically available metabolic imaging tools such as ^18^F-FDG-PET and ^99m^Tc-sestamibi SPECT have limitations. Therefore, new approaches are required to assess this metabolism, and novel metabolic MRI techniques including hyperpolarised [1-^13^C]pyruvate MRI and deuterium metabolic imaging offer promising alternatives. These techniques are non-radioactive, demonstrate spatial metabolic heterogeneity, and can probe metabolic flux beyond tracer uptake. This review aims to explore the potential of metabolic MRI in the clinical management of RCC by (1) summarising current clinical guidelines; (2) reviewing metabolic heterogeneity across RCC subtypes; (3) discussing the potential of metabolic MRI to advance the understanding of in vivo metabolism; (4) and finally suggesting future directions for research in this field.

## Simple Summary

Renal cell carcinoma (RCC) is the most common kidney cancer with an increasing incidence and mortality. Current diagnostic methods are limited, leading to delayed treatment and suboptimal patient outcomes. RCC subtypes have distinct metabolic phenotypes which have the potential to be used for better diagnosis and treatment. A number of emerging metabolic MRI techniques offer promising non-invasive alternatives to conventional imaging for assessing these differences across tumours, including hyperpolarised [1-^13^C]pyruvate MRI and deuterium metabolic imaging. These techniques provide potentially valuable information about tumour metabolism, which could be used for earlier detection, more accurate diagnosis, and improving treatment response monitoring. This review explores the potential of metabolic MRI in the clinical management of RCC and outlines future research directions.

## 1. Introduction

Renal cell carcinoma (RCC) is the most common kidney malignancy, with a steadily increasing incidence globally but with mortality rates that are not declining despite improvements in clinical management [1,2]. However, current diagnostic methods have limitations, including challenges in differentiating benign from malignant localised renal tumours and an inability to accurately detect early and successful response to treatment [3,4]. This can result in the delayed treatment of aggressive cases [5] and unnecessary anxiety for patients who ultimately are found to have benign lesions [6]. Biopsies are commonly used to assess tumour histology and aggressiveness but are invasive, not possible in all patients, and provide only a limited and potentially unrepresentative sample of the entire tumour mass [7]. Moreover, even after curative surgery, the disease may recur in up to 30% of patients, highlighting the need for the development of neoadjuvant and adjuvant treatment strategies, as well as better tools for surveillance and treatment response monitoring [8,9].

Different histological subtypes of renal cell tumours are known to manifest distinct metabolic alterations, particularly involving glucose metabolism, due to characteristic driver mutations. The most well-known example is the shift to glycolysis, also termed the Warburg effect [10]: in clear cell renal cell carcinoma (ccRCC), this effect occurs due to the loss of the von Hippel–Lindau (VHL) tumour suppressor gene, stabilising hypoxia-inducible factors (HIFs) and thereby activating expression of glycolytic genes [11,12]. Similarly, upregulated glycolysis is also found in the other two most common subtypes of RCC: papillary RCC (pRCC) and chromophobe RCC (chRCC) [13,14]. Changes in metabolism are also characteristic of the histological subtypes occurring due to loss-of-function in some tricyclic acid (TCA) cycle enzymes: succinate dehydrogenase-deficient (SDHd) RCC [15] and fumarate hydratase-deficient (FHd) RCC [16]. Suppressed metabolism is also present in the most common benign kidney tumour, renal oncocytoma (RO), due to a loss of mitochondrial complex I, leading to an accumulation of defective mitochondria and HIF destabilisation [17,18]. Considering these characteristic metabolic changes, an unanswered question is whether non-invasive metabolic imaging techniques can be used to better characterise kidney tumours and therefore improve clinical management and outcomes, including differentiating subtypes, probing intratumoural metabolic heterogeneity, and detecting successful treatment response earlier.

Clinically available tools for the assessment of tumour metabolism include the use of the radiolabelled glucose analogue fluorine-18-labelled fluorodeoxyglucose in conjunction with positron emission tomography (^18^F-FDG-PET), but this is limited in the kidney due to renal excretion of the tracer and has not been successfully used to characterise kidney tumours [19]. An alternative radionuclide is ^99m^Tc-sestamibi which accumulates in tissues with abundant mitochondria and can be detected using Single-Photon Emission Computed Tomography (SPECT); this has shown promise in differentiating benign and malignant masses, but still fails to accurately differentiate a benign renal oncocytoma from chRCC (with a sensitivity of 89% and specificity of 67%, based on a recent systematic review [20]). Therefore, identifying small malignant renal tumours with high sensitivity and specificity remains an unmet clinical need [21]. In addition, both PET and SPECT expose patients to ionising radiation and cannot discriminate individual downstream metabolites [19,20].

In comparison, metabolic MRI methods are powerful non-radioactive and non-invasive methods used to detect individual metabolite levels in vivo and intratumoural metabolic heterogeneity [22,23]. Proton MR spectroscopy (^1^H-MRS) is the most widely used approach but this largely assesses only steady-state metabolism rather than metabolic flux and can be susceptible to magnetic field inhomogeneity and motion artifacts [24,25,26,27]. In this review, we provide a rationale for how emerging metabolic MRI techniques, including hyperpolarised [1-^13^C]pyruvate MRI (HP ^13^C-MRI) and deuterium metabolic imaging (DMI), can be harnessed to guide clinical decisions in kidney cancer. State-of-the-art clinical guidelines will be summarised followed by evidence for metabolic heterogeneity in kidney tumours. Finally, perspectives for the use of metabolic MRI to advance the understanding of in vivo metabolism will be provided, as well as for its potential integration into the clinical management of localised kidney cancer, suggesting directions for future research.

## 2. Epidemiology, Pathology, and Current Clinical Guidelines

### 2.1. Epidemiology and Aetiology

Renal cell carcinoma (RCC) accounts for approximately 4% of all malignancies globally, representing the sixth and tenth most frequent cancer in women and men, respectively [28]. The incidence of RCC has increased significantly during the last two decades and currently accounts for >400,000 new cases per year globally [1]. While most lesions are localised renal masses, metastatic disease is detected in up to 17% of patients at diagnosis [29]. Detection of RCC at an advanced stage significantly reduces the 5-year cancer-specific survival (CSS), which ranges from 91% for low-risk localised tumours to 0–32% in late-stage disease [30]. Mortality rates therefore remain high, with 179,368 deaths globally in 2022, resulting in RCC being one of the most lethal urological malignancies [1,28]. RCC occurs more frequently in older and male patients, with risk factors including smoking, obesity, hypertension, end-stage renal failure, and acquired cystic kidney disease [28].

### 2.2. Histopathology

Tumours originating from renal tubular epithelium represent 75–80% of all kidney cancers [31] and encompass a heterogeneous group of histopathological types described by the 2022 World Health Organisation (WHO) classification under ICD-O-3.2 as “Renal cell tumours” [32]. The most common malignant subtypes are clear cell, papillary, and chromophobe (ccRCC, pRCC, chRCC), and the most common benign subtype is the oncocytoma (RO), with an incidence of up to 18% in tumours of <4 cm at presentation [33]. The metabolic nature of the driver mutations in SDHd-RCC and fumarate hydratase-deficient FHd-RCC results in these subtypes being of particular interest for the exploration of metabolic heterogeneity. RCC subtypes arise from distinct cells of origin along the nephron and are distinguished based on their histological morphology and molecular staining profile, which give rise to their nomenclature, as summarised in Table 1.

In addition to tumour subtype, histological evaluation includes tumour grading based on the World Health Organisation and International Society of Urological Pathology systems (WHO/ISUPs; for ccRCC and pRCC), sarcomatoid or rhabdoid differentiation, the presence of necrosis, microscopic lymphovascular invasion, surgical margins, and conventional pathological staging of the tumour, nodes, and metastases (pTNM) [30].

### 2.3. Diagnostic Evaluation

In the majority of patients (60%), RCCs are detected incidentally [42]. The classic triad of flank pain, macroscopic haematuria, and a palpable abdominal mass is rare and a sign of advanced disease [43]. Physical examination and laboratory investigations only play a limited role in diagnosis, although they have prognostic value for treatment stratification [43].

Imaging is central to clinical decision-making including detection, diagnosis, and characterisation of lesions at staging and follow-up. The Royal College of Radiologists in the UK [44] recommends imaging with CT of the chest, abdomen, and pelvis following contrast-medium imaging as the imaging of choice to evaluate the primary tumour and metastatic disease. However, standard-of-care imaging does not differentiate RCC from RO [4]. MRI is indicated for the evaluation of renal masses with indeterminate CT findings, typically small solid renal masses. The protocol of the Society of Abdominal Radiology is outlined in Table 2 [45,46].

However, characterisation of renal mass subtypes on MRI remains a clinical challenge, particularly for the overlapping features of RO and chRCC, and requires further development [47]. Recently, a five-tier Likert scoring algorithm for the likelihood of a renal mass being ccRCC on MRI was proposed which showed good sensitivity (75%) and specificity (78%) with a score of 4 or 5 [48,49].

Imaging assesses primary tumour staging based on the TNM system, which influences treatment decisions [50]. This includes assessment of tumour size, invasion of lymph nodes and adjacent structures (liver, spleen, muscles), extension into the venous system, and spreading into the lungs and adrenal glands.

Renal mass biopsy (RMB) is currently the gold-standard for determining the histology of radiologically indeterminate renal masses in patients who are candidates for active surveillance or ablative therapy, as well as for choosing the most appropriate targeted therapy in metastatic disease [31,43]. However, RMB is an invasive procedure which is not appropriate for some tumours due to their location, the biopsy results take time to process and may be non-diagnostic in up to 20% of first attempts, and the sample may be unrepresentative of the whole tumour due to heterogeneity, resulting in undergrading of the lesion in up to 16% of cases [5,7].

### 2.4. Treatment Strategies

Surgical excision with curative intent is the gold-standard treatment for localised and locally advanced tumours which are suspected to be RCC, while ablative techniques and active surveillance (AS) are recommended for histological subtypes with a lower risk of progression such as oncocytic renal neoplasms [2,43].

Partial nephrectomy is the surgical treatment of choice for organ-confined T1a tumours in patients with RCC of any tumour size with compromised kidney function, solitary kidneys, or bilateral tumours. Equally, ablative techniques can be applied as an alternative and preferred option in patients with cortical tumours ≤ 3 cm, in frail/comorbid patients, and in hereditary RCCs. Minimally invasive (laparoscopic or robot-assisted) radical nephrectomy is a recommended option for T2 tumours, while locally advanced tumours (T3 and T4) should be treated with an open radical nephrectomy approach (other than in selected patients in centres with high levels of robotic surgery expertise) with excision of the thrombus in cases of venous involvement, as well as any lymph node dissection of clinically enlarged lymph nodes for the purpose of staging, prognosis, and follow-up [2,43].

After surgery with curative intent, approximately 30% of patients experience recurrence [8,43]. Therefore, neoadjuvant and adjuvant treatment strategies for localised and locally advanced RCC are being currently investigated in clinical trials [30,43]. To date, no significant survival benefit has been identified for adjuvant therapy in randomised clinical trials, including using tyrosine kinase inhibitors (TKIs) [51,52,53], mTOR inhibitors [54], or the CAIX inhibitor girentuximab [55]. Although sunitinib improved disease-free survival (DFS) in a single randomised controlled trial, it has not been approved by the European Medicines Agency (EMA) due to high-grade toxicities [30]. Immune checkpoint inhibitors have shown a survival benefit in the metastatic setting, and although there is conflicting evidence from several adjuvant checkpoint inhibitor clinical trials, the Keynote 564 study demonstrated a DFS and an overall survival (OS) advantage of 1 year with adjuvant pembrolizumab therapy [56].

Optimal clinical trial designs for neoadjuvant studies are an area of high priority within the field currently, as these strategies have the potential to treat high-risk patients at the earliest possible opportunity [57]. Although prolonged surgical waiting times decrease patient survival, they may present an opportunity for the development of neoadjuvant treatment strategies [58,59]. To date, only a small number of phase II trials have reported results for TKIs and immune checkpoint inhibitors in the neoadjuvant setting [60], and further clinical trials are ongoing [61,62].

### 2.5. Follow-Up

Prognostic models can be used to assist with follow-up planning and predicting long-term outcomes. The Leibovich model is frequently used to predict the progression-free survival (PFS) and cancer-specific survival (CSS) of ccRCC, the most common histologic subtype [63,64]. A higher score denotes a greater progression risk, demanding more frequent follow-up investigations [63]. In addition, the histological subtypes also affect prognosis, with ccRCC showing the worst 10-year CSS compared to pRCC and chRCC after correction for tumour stage [34,43].

Follow-up imaging after surgery is used to identify postoperative complications, local/contralateral recurrence, the appearance of distant metastases, and related cardiovascular events. Postsurgical CT imaging of the thorax, abdomen, and pelvis every 3–6 months is recommended, with a higher frequency of imaging for high-risk RCCs. Objective response to systemic therapy in metastatic disease, as well as in the context of adjuvant and neoadjuvant treatment strategies, can be evaluated using the Response Evaluation Criteria in Solid Tumours (RECIST) determined on imaging. RECIST 1.1 is widely used to assess response in clinical studies [65]; however, these size changes do not necessarily correspond to a clinically validated endpoint [30], and measuring size alone may be insufficient to capture the response to some novel targeted therapies. Given the time interval required to elicit a significant size change, the RECIST are limited in the evaluation of short-term neoadjuvant therapies [9,65].

## 3. Metabolic Heterogeneity in Kidney Cancer

Kidney cancer is characterised by a high degree of heterogeneity on many different spatial scales including macroscopically, histologically, functionally, metabolically, and at the cellular level [66]. This heterogeneity may lead to problems in clinical management, such as sampling errors on RMB, requirements for combinational drug therapies, and a lack of accurate biomarkers to monitor the response to this treatment [43,67]. Not only is there significant variation in imaging between histological subtypes, but there is also heterogeneity within an individual tumour (intratumoural), between tumours in a single patient, and between patients with the same tumour subtype (intertumoural). Imaging is a key technology used to capture this spatial heterogeneity and how it changes over time and with treatment [68]. Kidney cancer is an exemplar of metabolic reprogramming, which describes the shift to alternative energy-yielding pathways to fuel tumour progression [69]. The best known example of such metabolic shifts is the Warburg effect, also termed aerobic glycolysis, whereby cancer cells preferably convert pyruvate to lactate rather than import it into mitochondria for oxidative phosphorylation [70]. Across different histological subtypes, specific genetic mutations underlie the occurrence of characteristic metabolic phenotypes, and this knowledge could be harnessed to aid in the differentiation of benign and malignant renal masses based on metabolism. In addition, kidney tumours exhibit high intratumoural heterogeneity, on both genetic and metabolic levels [71,72]. Understanding the biology of this intratumoural variation could enhance image-guided biopsies to the region with the highest chance of a diagnostic yield and with the most aggressive phenotype, as suggested by perfusion/metabolism mismatch studies in other cancer types [73,74,75]. The current status of research on the metabolic heterogeneity of glucose metabolism in kidney cancer is summarised below [76].

### 3.1. Intertumuoral Heterogeneity

The genetic alterations underpinning RCC are involved in many essential metabolic pathways, and these result in metabolic dysregulation including the Warburg effect [13]. The preferred use of glycolysis for energy generation in RCC, instead of the energetically more efficient oxidative phosphorylation pathway, supports the rapid proliferative demands of tumour growth and replenishes the NAD^+^ pool for lactate production [77]. In comparison, renal oncocytoma is a benign renal cell tumour and exhibits a downregulation of metabolism due to lower energetic demands for proliferation [18].

#### 3.1.1. Clear Cell RCC

In 90% of sporadic ccRCCs, there is loss of the VHL tumour suppressor gene, which controls cellular oxygen sensing via regulating the stability of HIFs. Loss of the VHL protein leads to the stabilisation and accumulation of HIFs and consequent activation of their transcriptional targets, causing a metabolic shift towards aerobic glycolysis, increased angiogenesis, and suppressed oxidative phosphorylation [13]. The utilisation of glucose in these tumours can be identified using isotopomer analysis in postoperative human ccRCC tissue samples following intraoperative infusion of ubiquitously labelled [U-^13^C_6_] glucose, and these approaches have demonstrated reduced labelling of TCA intermediates compared to the normal kidney [78].

#### 3.1.2. Papillary RCC

In pRCC, the most frequent mutation is near-universal activation of the MET oncogene; MET is a receptor tyrosine kinase which stimulates downstream pathways, inducing glycolysis, and reduces the expression of enzymes involved in gluconeogenesis, the TCA cycle, and oxidative phosphorylation [79].

#### 3.1.3. SDHd-RCC and FHd-RCC

Loss-of-function of TCA cycle enzymes is characteristic of SDHd-RCC and FHd-RCC, resulting in the accumulation of the oncometabolites succinate and fumarate, respectively [15], which in turn promotes tumorigenesis. Both metabolites stabilise HIFs which subsequently upregulate glycolysis and angiogenesis to meet the high energetic and biosynthetic demands of these aggressive tumour types [16].

#### 3.1.4. Chromophobe RCC and Renal Oncocytoma

In contrast to other RCCs, the somatic mutation rate in chRCC is low, and most cases present with a loss of heterozygosity (LOH) in chromosomes Y, 1, 2, 6, 10, 13, 17, and 21 [43]. Less consistently, activating mutations of the mTOR pathway are found which are known to promote biosynthesis and tumour survival [13]. The lack of a specific driver mutation suggests that tumorigenesis in this subtype may be due to other mechanisms, and indeed alterations in mitochondrial DNA (mtDNA) have been reported previously [17]. As with the benign RO, a characteristic feature of both tumour types is an abundance of mitochondria. However, the main difference between them is differential mtDNA mutations. chRCC shows a general mtDNA reduction rather than selective complex I mutations, which are the hallmark of RO [17]. Ablation of complex I destabilises HIF1a, causing overall downregulation of metabolic pathways in RO [18], and leads to an accumulation of defective mitochondria with impaired autophagy, limiting progression to malignancy [80,81]. On the other hand, it is less clear to what extent the mitochondria preserve functionality in chRCC, as reports so far have been contradictory: reduced expression of genes and metabolite concentrations involved in the TCA cycle and OXPHOS were reported in some studies [13,82], while n increased labelling of TCA cycle metabolites was observed in postoperative chRCC tissues after intraoperative infusion with [U-^13^C] glucose [83]. In contrast to RO, the glycolytic pathways in chRCC are upregulated, but not to the same extent as in ccRCC [82].

### 3.2. Intratumoural Heterogeneity

Studies assessing the intratumoural heterogeneity of RCC metabolism are only beginning to emerge and are largely limited to the most common subtype, ccRCC. The upregulation of glycolytic enzymes and metabolites increases with ccRCC tumour grade at the expense of TCA cycle intermediaries [84,85,86]. However, many of these studies analysed a single sample per patient and therefore cannot report on intratumoural heterogeneity. Okegawa et al. [71] have reported different metabolic phenotypes from spatially separated regions within the same primary tumour. By applying global metabolomic analysis on multiple regions from 18 patients and performing isotope tracer experiments on ex vivo tissue slices from 2 patients, they were able to identify two distinct metabolic clusters, differing in their abundance of glycolytic metabolites. Isotope tracer experiments with [U-^13^C]glucose indicated differential fractional enrichments of pyruvate and citrate across regions. Importantly, they identified altered pyruvate metabolism across all tumour sites, which could be exploited as a therapeutic vulnerability in the future. Further evidence for the role of pyruvate has been shown using a mitochondrial pyruvate carrier (MPC) inhibitor which supresses the growth of tumour cells in vitro and in vivo in patient-derived xenograft tumours in mice [71]. Insights from the immune microenvironment of ccRCC demonstrate that high glucose consumption and lactate production promote immunosuppression [87], and recent reports assessing the intratumoural heterogeneity of the immune landscape suggest a role of immunometabolism in driving the emergence of these intratumoural habitats [88,89]. Similarly, a study investigating intratumoural regions in ccRCC separated by high and low perfusion on contrast-enhanced MRI correlated these with varying immune transcriptomic signatures on biopsy, although they did not assess the role of metabolism [90].

Metabolic heterogeneity of kidney tumours has been studied extensively in vitro, as well as using ex vivo tissue samples and in preclinical RCC models [69]. However, in vitro and ex vivo metabolomic analyses cannot assess the dynamics of metabolism in vivo, while preclinical models cannot capture the tumour clonality which occurs in patients. In addition, due to the lack of preclinical models for rarer RCCs and for benign subtypes, these tumours remain understudied [69]. Clinical imaging tools which can detect metabolic alterations directly in patients circumvent the dependence on tissue extraction for analysis and therefore mitigate against the potential of perturbing the highly delicate metabolic networks during the sampling process.

## 4. Clinical Imaging of Metabolism in Kidney Cancer

### 4.1. Nuclear Imaging Techniques

#### 4.1.1. Positron Emission Tomography with [^18^F]Fluorodeoxyglucose ([^18^F]FDG-PET)

**Figure 1:**
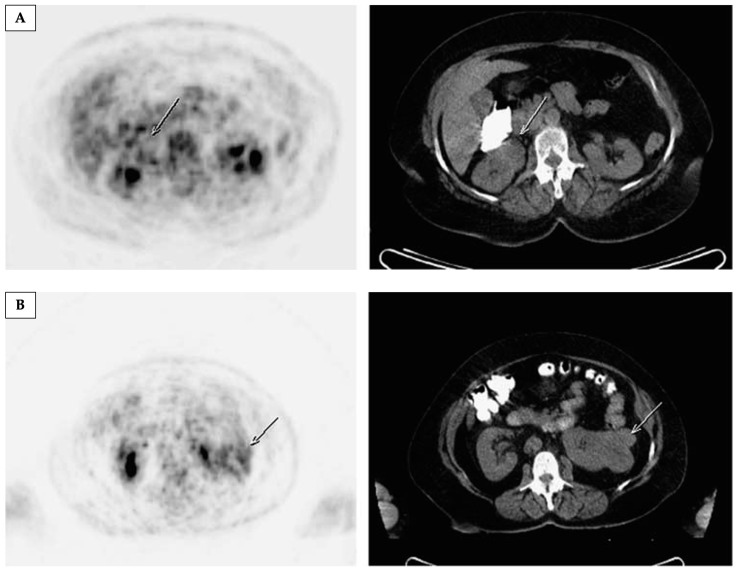
[^18^F]FDG-PET cannot accurately differentiate between malignant and benign renal masses. (**A**) Axial PET and CT images from a [^18^F]FDG-PET/CT study showing low-grade tracer uptake in a right renal mass (arrowed), which was later confirmed to be renal cell cancer. (**B**) Axial slices of PET and CT from a second patient showing some [^18^F]FDG uptake in a left renal mass (arrowed), which was later shown to be an oncocytoma. Reprinted, under a CC BY license (license number: 5962670886654) as mediated by the Copyright Clearance Center (Chicago, IL, USA), from reference [93].

Currently, the only routine clinical tool for the assessment of tumour metabolism uses a radiolabelled glucose analogue, 2-[^18^F]fluoro-2-deoxy-D-glucose ([^18^F]fluorodeoxyglucose, [^18^F]FDG), in conjunction with positron emission tomography (PET). While it enables imaging of glucose uptake and has a long history of clinical success in oncology [91], it is constrained by renal excretion of the tracer which can prevent an accurate assessment of uptake in the adjacent tumour, and thus has a limited role in characterising localised kidney tumours [19]. Nevertheless, the technique has been explored as a non-invasive imaging tool of metabolic dysregulation across different RCC subtypes. In a retrospective study of 147 patients, [^18^F]FDG uptake was significantly lower in chRCC compared to ccRCC and pRCC, as well as in low- compared to high-grade ccRCCs and pRCCs [92]. However, [^18^F]FDG-PET was unreliable in differentiating between RCC and RO, where more than half of malignant lesions were falsely negative on PET, and a single RO was a false positive [93], as shown in Figure 1.

A systematic review of the literature determined that [^18^F]FDG-PET may be a viable option for recurrent and metastatic RCC, where it can be used to monitor treatment response and predict survival [19]. A reduction in [^18^F]FDG uptake in metastatic lesions after a course of systemic therapy has been shown to correlate with longer survival rates [94,95,96,97] and can outperform the RECIST in early response evaluation of skeletal lesions after sorafenib in metastatic RCC [98].

#### 4.1.2. ^99^Tc-Sestamibi SPECT

**Figure 2:**
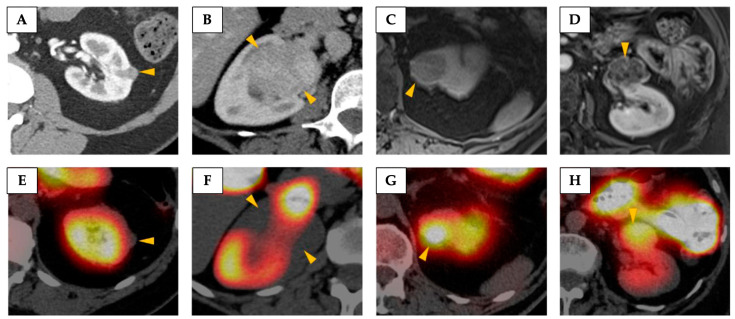
A demonstration of the high variability of ^99m^Tc-sestamibi uptake in chromophobe renal cell carcinomas (chRCCs). Axial images from contrast-enhanced CT (**A**,**B**) and post-contrast T1-weighted fat-saturated MRI (**C**,**D**) showing representative images of the four chRCCs included in the study performed by Rowe et al. [99]. (**E**–**H**) Corresponding ^99m^Tc-sestamibi SPECT/CT images of the same tumours demonstrating the highly variable uptake seen in this tumour type. Tumours are denoted by yellow arrowheads. The tumours in (**A**/**E**) and (**B**/**F**) were qualitatively considered cold, whereas the tumours in (**C**/**G**) and (**D**/**H**) were qualitatively considered hot. Reprinted, under a CC BY 4.0 license, from reference [99].

Another emerging clinical imaging technique that can exploit metabolic alterations in renal masses is ^99m^Tc-sestamibi Single-Photon Emission Computed Tomography (SPECT) [21]. ^99m^Tc-sestamibi accumulates in tissues with high mitochondrial content, which is a characteristic of oncocytic tumours [99]. While still under investigation as a diagnostic tool to evaluate indeterminate renal masses, the results showed promise in discriminating between oncocytic renal tumours and malignant masses [21]. However, chRCC is also characterised by mitochondrial proliferation, and a meta-analysis has revealed a low specificity of ^99m^Tc-sestamibi SPECT in differentiating between oncocytic renal tumours and chRCC [21]. Figure 2 demonstrates the high variability of ^99m^Tc-sestamibi uptake in chRCCs.

In summary, the role of [^18^F]FDG-PET and ^99m^Tc-sestamibi SPECT as potential routine clinical tools in kidney cancer management remains limited. In addition, both techniques have several other drawbacks, including radiation exposure, long scanning times of up to two hours, and an inability to probe metabolism beyond glucose uptake and mitochondrial accumulation [27,99], and therefore do not directly report on the balance between the Warburg effect and oxidative phosphorylation in the tissue of interest.

### 4.2. Imaging Metabolism Using Magnetic Resonance

#### 4.2.1. Magnetic Resonance Spectroscopy (MRS)

Proton MR spectroscopy (^1^H-MRS) is based on the principles of nuclear magnetic resonance and offers a non-radioactive alternative to detect tissue metabolic signatures in vivo. The method probes metabolites at a high concentration due to the low sensitivity compared to PET, is highly susceptible to magnetic field inhomogeneity and motion artifacts, and has a low spatial resolution, which limits its role in evaluating metabolic heterogeneity [26]. Studies investigating the role of this technique in characterising renal masses have observed an increase in both choline and lipid concentrations in malignant compared to benign lesions and with increasing RCC grade [100,101,102]. However, metabolites involved in oxidative metabolism are largely below the limit of detection, with the exception of elevated fumarate in HLRCC syndrome-associated renal tumours [24] and in FHd-RCC [25,103]. However, metabolic MRI approaches could provide important tools to study intratumoural heterogeneity in the absence of ionising radiation. New complementary spectroscopic approaches are being developed which are robust to magnetic field disruptions and motion artifacts, detect metabolic conversions beyond glucose uptake, and provide increased spatial resolution [27,104]. Below, we describe two techniques which have been introduced into the clinical setting and have recently been applied to assess metabolism in renal tumours.

#### 4.2.2. Hyperpolarised [1-^13^C]Pyruvate MRI

Hyperpolarised [1-^13^C]pyruvate MRI is an emerging clinical imaging technique based on the intravenous injection of non-radioactive carbon-13 (^13^C)-labelled pyruvate after it has undergone a process termed hyperpolarisation; this process significantly increases the sensitivity for detection, therefore facilitating tissue metabolism to be detected in real time and non-invasively. Hyperpolarisation refers to a transient increase in nuclear spins aligned with the main magnetic field, which can be achieved via several methods. Dynamic nuclear polarisation (DNP) is the current leading approach for clinical applications and involves mixing ^13^C-pyruvate with an electron-rich compound or free radical before placing this sample into the extreme physical conditions of a strong magnetic field (3–7 T) close to absolute zero (1 K) while irradiating with microwaves for 1–2 h. The resulting effect is the transfer of electron polarisation to the ^13^C-nuclei in pyruvate, which significantly amplifies the signal-to-noise ratio (SNR) of the hyperpolarised ^13^C-pyruvate on MR spectroscopy and imaging more than 10,000-fold, thus facilitating the detection of metabolism [27]. This offers a non-invasive way to detect dynamic metabolism through the conversion of hyperpolarised ^13^C-pyruvate into other metabolites, including ^13^C-lactate as a measure of the Warburg effect and ^13^C-bicarbonate as a biomarker of entry into the TCA cycle [105]. The enhanced signal from hyperpolarisation is sufficient to image for several minutes but this decreases rapidly over time due to relaxation effects and the irretrievable loss of signal as the images are acquired, and therefore, an on-site hyperpolariser is required. However, the short acquisition times that this necessitates are beneficial for clinical translation and are enabled by rapid imaging sequences [106,22].

The first clinical study was published in 2013, showing that prostate cancer exhibited a higher pyruvate-to-lactate conversion compared to surrounding normal tissue [107]. In imaging of kidney cancer, HP ^13^C-MRI has shown promise preclinically, where lactate production has been identified as a biomarker for tumour aggressiveness in RCC cell lines and orthotopic murine tumours [108,109] and is increased in patient-derived ccRCCs compared to benign kidney tumours (two ROs and one angiomyolipoma), incubated in a tissue culture bioreactor [110]. This difference was shown to be due to an increase in a subunit of the enzyme lactate dehydrogenase (LDHA), which catalyses the exchange reaction between pyruvate and lactate, and the lactate exporter or monocarboxylate transporter 4 (MCT4) [110]. Both LDHA and MCT4 are targets for HIF transcriptional programs and have prognostic value in RCC, where upregulation predicts worse survival outcomes [111,112].

**Figure 3:**
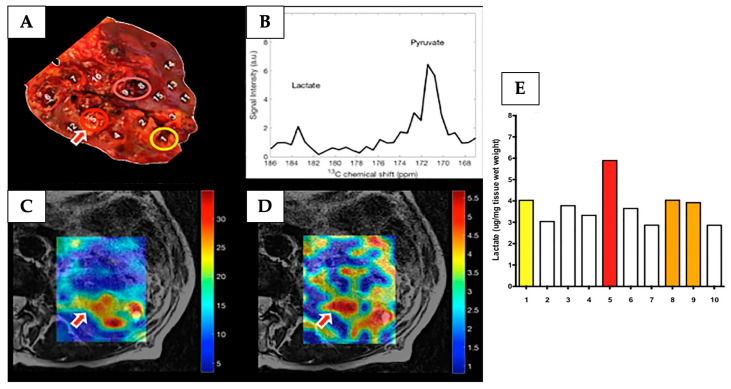
HP ^13^C-MRI detected intratumoural metabolic heterogeneity in a patient with ccRCC. (**A**) The post-nephrectomy kidney axially sliced at the level of the renal hilum from where 15 tissue samples were collected (1–10 tumour samples and 11–15 non-tumour samples). (**B**–**D**) Images from HP ^13^C-MRI acquisition: (**B**) the corresponding spectra of the arrowed region “5” on (**A**) image. The spectrum shows peaks corresponding to lactate and pyruvate. (**C**) An interpolated HP ^13^C-pyruvate map overlaid on a T2w image. (**D**) An interpolated HP ^13^C-lactate map overlaid on a T2w image. (**E**) A bar chart of liquid chromatography–mass spectrometry analysis of the multiregional samples from the tumour. The highest level of lactate accumulation was found in region “5” (red bar), consistence with the findings of the HP ^13^C-lactate map in (**D**). Other coloured bars correspond to coloured circles in (**A**). Reprinted, under a CC BY 4.0 license, from reference [113].

Clinical kidney cancer imaging using HP ^13^C-MRI has reported intratumoural metabolic heterogeneity in a single case of ccRCC [113]. The region with the highest ^13^C-lactate signal corresponded to the highest lactate as determined via mass spectrometry (MS) on postoperative tissue sample analysis, as shown in Figure 3.

**Figure 4:**
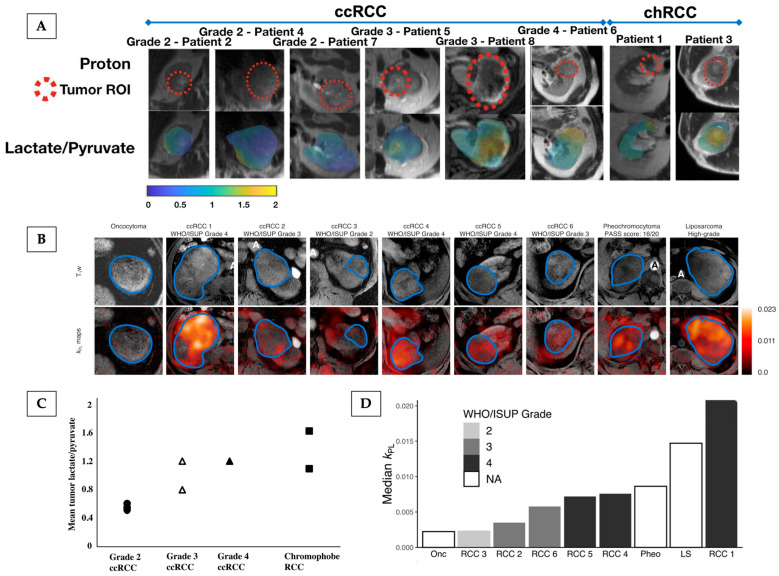
HP ^13^C-MRI demonstrating intertumoural heterogeneity in kidney cancer, showing the potential to probe kidney tumour aggressiveness. (**A**,**B**) depict anatomical and HP ^13^C-MRI images from patients bearing renal masses, outlined with a dashed red line in (**A**) and blue line in (**B**), who were recruited to studies performed by Tang et al. [114] (**A**) and Ursprung et al. [115] (**B**). Tang et al. [114] measured the HP ^13^C-lactate/pyruvate ratio, while Ursprung et al. [115] calculated the *k*_PL_ as measures of pyruvate-to-lactate metabolism. (**C**) A dot plot representing the mean tumour lactate/pyruvate ratio in the 8 patients stratified by tumour histology and grade, as illustrated in (**A**). There is a trend toward higher lactate/pyruvate ratios in high-grade (grades 3 and 4) ccRCCs compared with low-grade (grade 2) ccRCCs. Both chRCCs demonstrate a relatively high mean tumour lactate/pyruvate ratio; the chRCC with the highest lactate/pyruvate ratio had a pathologic finding of microscopic necrosis associated with aggressive biology. (**D**) Bar plots representing the median *k*_PL_ across WHO/ISUP grades, where increasing tumour grade was associated with increased metabolic activity in ccRCC. The phaeochromocytoma and liposarcoma showed metabolic activity comparable to grade 4 ccRCC. The benign renal oncocytoma showed the lowest metabolic activity. ccRCC = clear cell renal cell carcinoma; chRCC = chromophobe renal cell carcinoma; *k*_PL_ = apparent exchange rate constant for pyruvate-to-lactate conversion; LS = liposarcoma; Onc = oncocytoma; Pheo = phaeochromocytoma; RCC = renal cell carcinoma; WHO/ISUP = World Health Organisation/International Society of Urological Pathology. Reprinted, under a CC BY 4.0 license from reference [115], and under a CC BY license (License number: 5961980546793) as mediated by Copyright Clearance Center, from reference [114].

Tang et al. [114] imaged 11 patients with kidney tumours, in which a trend towards a higher lactate/pyruvate ratio in high-grade compared to low-grade ccRCCs was demonstrated, as well as the highest lactate/pyruvate ratio being present in the aggressive type of chRCC. Ursprung et al. [115] showed that ^13^C-lactate labelling could be used to distinguish high-grade ccRCC from lower-grade tumours, and that lactate labelling correlated with the pyruvate transporter (MCT1), which was shown to be a predictor of overall and disease-free survival. Furthermore, a case of a renal oncocytoma displayed the lowest pyruvate-to-lactate conversion compared to a range of malignant masses, including ccRCCs, suggesting that HP ^13^C-MRI may be a potential clinical imaging tool for discriminating benign from malignant kidney tumours [115]as demonstrated in Figure 4.

The technique has also shown promise in treatment response monitoring. While this has been unexplored in RCC so far, ^13^C-lactate generation was identified as a potential biomarker of treatment response in other cancers. In prostate and breast cancer, a decrease in tumour ^13^C-lactate labelling was demonstrated after several weeks of treatment [116,117]. Furthermore, the technique detected responses to neoadjuvant therapy in breast cancer only 7–11 days after treatment and has outperformed conventional proton MRI in distinguishing responders from non-responders [118].

While findings from initial clinical studies on HP ^13^C-MRI are highly promising, several challenges need to be addressed before it can be used as a routine clinical imaging tool. Currently, the technique requires a dedicated pharmacy facility, bespoke consumables (termed pharmacy kits), and a team of trained professionals with a broad range of skills, and overall remains expensive. However, there are currently ongoing efforts to address these technical challenges, such as the development of new polarisation methods with improved ease of use and reliability, centralisation of consumable filling, and the development of standardised acquisition and analysis methods. Finally, clinical studies which enrol larger patient cohorts and compare the technique to standard clinical imaging could provide the evidence required for the potential use of HP ^13^C-MRI as a routine clinical tool [119].

#### 4.2.3. Deuterium Metabolic Imaging

**Figure 5:**
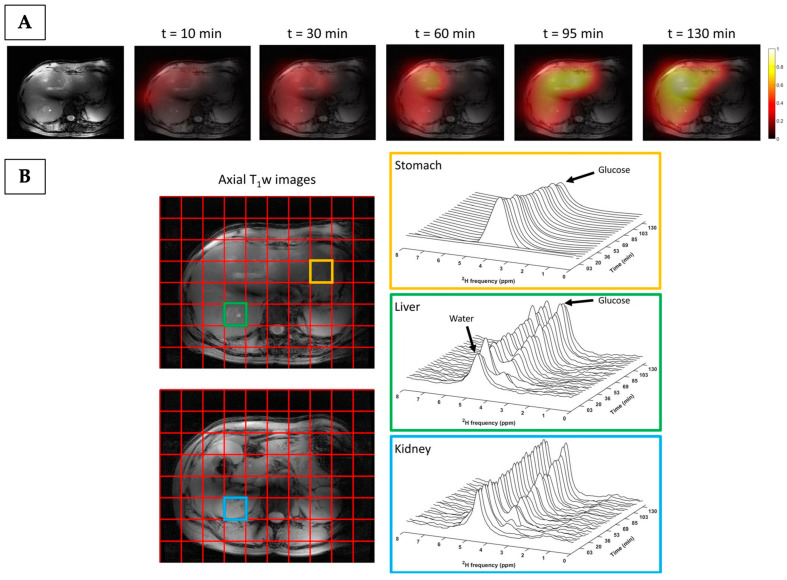
Deuterium metabolic imaging of the stomach, liver, and kidney in a healthy volunteer, acquired between 0 and 130 min after oral administration of [6,6′-^2^H_2_]glucose. (**A**) Axial T1-weighted images and maps of deuterated glucose signal overlaid on anatomical MRI images across the time course. The deuterated glucose signal increases over time. (**B**) The DMI grid overlaid on axial T1-weighted images, with annotated voxels of interest located in the stomach (orange), liver (green), and right kidney (blue). The corresponding deuterium spectra acquired over time and extracted from each of the voxels are shown. In the stomach, the glucose signal was observed immediately after intake and slowly decayed thereafter. In comparison, the deuterated glucose signal (and to a lesser extent water signal) increased over time in voxels located within the liver and kidney. Reprinted, under a CC BY 4.0 license, from reference [124].

More recently, deuterium (^2^H) MRS imaging (also termed deuterium metabolic imaging or DMI) has been introduced as a novel metabolic imaging technique, with the first-in-human study published in 2018 [120]. The technique uses endogenous ^2^H-labelled probes such as [6,6′-^2^H_2_]glucose to non-invasively detect tissue metabolism using spectroscopic imaging. The first study was performed at a high field strength (7 T) by De Feyter et al., who imaged a patient with glioblastoma after oral administration of [6,6′-^2^H_2_]glucose, detecting increased labelling of lactate and decreased labelling of the combined glutamate/glutamine peak (Glx as a surrogate measure for TCA flux) in the tumour compared to the adjacent normal-appearing brain parenchyma [120]. This finding was confirmed in a small cohort of patients with brain tumours of varying aggressiveness [121], and subsequent studies have shown the feasibility of the technique at clinical field strength (3 T) for neurological imaging [122,123]. It has recently been translated to abdominal imaging to assess ^2^H-glucose uptake and metabolism in the liver and kidney [124,125,126], as depicted in Figure 5.

Deuterium metabolic imaging offers several advantages as a potential clinical imaging tool compared to other spectroscopic methods: the probes can be administered orally which is preferential for patients, deuterium has a low natural abundance which alleviates the need for the water and lipid suppression that is often required in ^1^H-MRS, deuterium has a short relaxation time which allows for signal averaging that enhances sensitivity and facilitates imaging over longer timescales [127,128], and DMI has lower technology costs compared to hyperpolarisation methods. However, the presence of the dominant signal from oral ^2^H-glucose within the stomach can restrict the abdominal field-of-view and affect quantification [126,129], and this will need to be addressed if DMI is to be applied more widely for non-invasive imaging of glucose metabolism in renal cancer.

## 5. Conclusion and Future Direction

Management of patients with localised kidney tumours has several unmet clinical challenges, including the low specificity of standard-of-care clinical imaging for assessing the aggressiveness of an incidental renal mass, the sampling error and limitations of renal mass biopsy, the lack of predictive clinical biomarkers, and the availability of methods to monitor early treatment response accurately. The metabolic reprogramming that is characteristic of different renal tumour subtypes, which in turn correlates with aggressiveness, offers an opportunity to better stratify patients. In addition, the marked intertumoural and intratumoural heterogeneity of kidney tumours is ideally probed using non-invasive clinical imaging methods. Therefore, metabolic MRI techniques provide an opportunity to probe both metabolic dysregulation and its heterogeneity, with the possibility of improving patient morbidity and mortality, as well as reducing the economic burdens of surgery and anticancer therapeutics [130]. While the current high costs prevent widespread adoption of this technology into clinical workflow today, these costs are likely to reduce with the introduction of new approaches for hyperpolarisation. In time, these costs may become similar to those incurred for utilising PET and can be offset by the potential savings of using imaging to replace expensive therapeutics that are ineffective, which represents a significant burden on healthcare systems [130]. Therefore, metabolic MRI techniques have a promising future in renal cancer research and could address some of the unmet clinical needs in this field (Table 3).

## Figures and Tables

**Table 1:** A summary of the main renal tumour subtypes and their characteristics.

Renal Tumour Subtype	Characteristics
Clear cell RCC (ccRCC)	- The most common subtype, representing 80% of renal cell tumours, arising from epithelial cells in the proximal convoluted tubule [31]. - Often occurs sporadically but can be present as a part of hereditary syndromes, such as VHL, tuberous sclerosis complex (TSC), or BAP1 tumour predisposition [34]. - Microscopically, cells typically present with lipid-rich (“clear”) cytoplasm, embedded in an intricate network of capillary vessels. Characteristic immunohistochemical staining is strongly positive for carbonic anhydrase 9 (CAIX) [35].
Papillary RCC (pRCC)	- The second most common subtype (10–15% of RCCs) [36], arising from the S3 segment of the proximal convoluted tubule [37]. - Most often occurs sporadically but can also be found as part of a genetic syndrome such as familial pRCC syndrome resulting from mutations in the MET gene. - Macroscopically appears solid, grey/brown in colour, and shows frequent necrosis and haemorrhage. Microscopically characterised by cells organised into papillary structures [32,38].
Chromophobe RCC (chRCC)	- The third most common subtype (3–5% of all RCCs) [34], occurring sporadically or as part of the Birt–Hogg–Dubé (BHD) cancer syndrome, resulting from genetic aberrations in the FLCN gene. - Macroscopically appears mahogany brown in colour, hypovascular, and as a well-demarcated mass often with a central scar [34,39]. - The cells of origin are the intercalated cells of the distal tubule and collecting duct [37]. Typically, chRCC presents with nuclear atypia in large cells with prominent cell membranes and either pale or eosinophilic (ballooned, oncocytoma-like cells) cytoplasm [34,39].
Renal Oncocytoma (RO)	- It has an important differential diagnosis with chRCC due to their morphological and histological similarities, but they have different treatment pathways: RO is a benign entity, monitored by active surveillance, while chRCC is malignant and therefore prioritised for surgery [34,40]. - Conventional immunohistochemistry (IHC) staining patterns can overlap between the two tumours, with the term oncocytic tumours being an emerging entity [32,41].
SDHd-RCC and FHd-RCC	- Rare tumour subtypes which result from tumorigenesis secondary to inactivation of the SDH and FH enzymes, respectively, often due to germline mutational losses. - Both tumours can be identified on IHC by their characteristic negative staining for their respective enzymes and should be prioritised for treatment due to their highly aggressive behaviour [32,34].

**Table 2:** MRI renal mass protocol. Source: Society of Abdominal Radiology (2018) [45].

Sequence	Plane	Slice Thickness/Gap	Comments
2D T2w SSFSE	Axial/Coronal	Axial: 4–5 mm/no gapCoronal: 5–6 mm/no gap	Alternative: 2D axial T2w FSE
2D T1w GRE in/out phase	Axial	5–6 mm/0.5–1 mm	Alternative: 3D Dixon, 3–4 mm/no gap
3D T1w SPGR fat saturation	Axial/Coronal	3–4 mm/no gap	
3D dynamic T1w SPGR fat saturation, 0.1 mL/kg of 1M Gd contrast	Axial/Coronal	3–4 mm/no gap	30, 90–100, and 180–210 s, subtraction imaging; after dynamic series, obtain the other plane at 240 s
**Optional sequences**			
3D T1w SPGR fat saturation	Axial/Coronal	3–4 mm/no gap	5–7 min post-contrast, image in plane perpendicular to the dynamic acquisition
Diffusion-weighted imaging (DWI)	Axial	5–6 mm/no gap	b-values: 0–50, 400–500, 800–1000 s/mm^2^

DWI = diffusion-weighted imaging; FSE = fast spin echo; GRE = gradient echo; SPGR = spoiled gradient; SSFSE = single-shot fast spin echo.

**Table 3:** Outlook: suggestions for future research directions.

Unmet Clinical Need	Possible Applications of Metabolic MRI and the Research Required to Assess These Applications
Sampling error of renal mass biopsy	Apply HP ^13^C-MRI to assess intratumoural metabolic variation and to enable biopsies to be targeted to the most aggressive tumour subregions [131,132]
Differentiating benign and malignant renal tumour subtypes	Undertake large multicentre HP ^13^C-MRI studies to assess metabolism in a range of renal tumour subtypes
	Assess the role of DMI to characterise benign and malignant renal tumours
Validated biomarkers for treatment response monitoring	Apply HP ^13^C-MRI to characterise metabolic response to neoadjuvant treatment of RCC as well as in the metastatic setting [133]
Biological validation of metabolic MRI	Validate metabolic MRI methods against tissue measures of metabolism to determine the biological mechanisms influencing metabolic imaging phenotypes
Clinical validation of metabolic MRI	Assess the added value of metabolic MRI over current standard-of-care imaging methods for probing intratumoural heterogeneity, determining tumour aggressiveness, targeting biopsies, and assessing response to therapy

## Data Availability

Not applicable.

## Abbreviations

AS	active surveillance
BHD	Birt–Hogg–Dubé
CA	carbonic anhydrase
CAIX	carbonic anhydrase 9
chRCC	chromophobe renal cell carcinoma
CSS	cancer-specific survival
DFS	disease-free survival
DMI	deuterium metabolic imaging
DNP	dynamic nuclear polarisation
DWI	diffusion-weighted imaging
EMA	European Medicines Agency
FDG-PET	fluorine-18-labelled fluorodeoxyglucose in conjunction with positron emission tomography
FHd-RCC	fumarate hydratase-deficient renal cell carcinoma
FSE	fast spin echo
Glx	glutamine/glutamate
GRE	gradient echo
HIF	hypoxia-inducible factor
HLRCC	hereditary leiomyomatosis and renal cell cancer
HP ^13^C-MRI	hyperpolarised [1-^13^C]pyruvate MRI
IHC	immunohistochemistry
LDHA	lactate dehydrogenase A
MCT1	monocarboxylate transporter 1
MPC	mitochondrial pyruvate carrier
MRS	magnetic resonance spectroscopy
mtDNA	mitochondrial DNA
OS	overall survival
OXPHOS	oxidative phosphorylation
PDH	pyruvate dehydrogenase
PFS	progression-free survival
pRCC	papillary renal cell carcinoma
pTNM	pathological tumour node metastasis staging
RCC	renal cell carcinoma
RECIST	Response Evaluation Criteria in Solid Tumours
RMB	renal mass biopsy
RO	renal oncocytoma
SDHd-RCC	succinate dehydrogenase-deficient renal cell carcinoma
SNR	signal-to-noise ratio
SPECT	Single-Photon Emission Computed Tomography
SPGR	spoiled gradient
SSFSE	single-shot fast spin echo
TCA	tricarboxylic acid
TKI	tyrosine kinase
TSC	tuberous sclerosis complex
VHL	von Hippel–Lindau
WHO	World Health Organisation
